# Use of sodium 4-phenylbutyrate to define therapeutic parameters for reducing intracerebral hemorrhage and myopathy in *Col4a1* mutant mice

**DOI:** 10.1242/dmm.034157

**Published:** 2018-07-04

**Authors:** Genki Hayashi, Cassandre Labelle-Dumais, Douglas B. Gould

**Affiliations:** Department of Ophthalmology, Department of Anatomy, Institute for Human Genetics, University of California, San Francisco, CA 94143-0730, USA

**Keywords:** Cerebral small vessel disease, Stroke, Intracerebral hemorrhage, Collagen, COL4A1, Myopathy, Drug therapy, Chaperones

## Abstract

Collagen type IV alpha 1 (COL4A1) and alpha 2 (COL4A2) form heterotrimers that constitute a major component of nearly all basement membranes. *COL4A1* and *COL4A2* mutations cause a multisystem disorder that includes variable cerebrovascular and skeletal muscle manifestations. The pathogenicity of *COL4A1* and *COL4A2* mutations is generally attributed to impaired secretion into basement membranes. Sodium 4-phenylbutyrate (4PBA) is a US Food and Drug Administration-approved drug that promotes mutant heterotrimer secretion *in vitro* and *in vivo*. Here, we use different 4PBA treatment paradigms to define therapeutic parameters for preventing cerebrovascular and muscular pathologies in *Col4a1* mutant mice. We show the efficacy of long-term 4PBA treatment in reducing the severity of intracerebral hemorrhages (ICHs) in *Col4a1* mutant mice aged up to 8 months. In addition, we demonstrate that maximal efficacy of 4PBA on ICH and myopathy was achieved when treatment was initiated prenatally, whereby even transient 4PBA administration had lasting benefits after being discontinued. Importantly, postnatal treatment with 4PBA also reduced ICH and skeletal myopathy severities in *Col4a1* mutant mice, which has significant clinical implications for patients with *COL4A1* and *COL4A2* mutations.

This article has an associated First Person interview with the first author of the paper.

## Abstract (precis)

**Summary:** *COL4A1* mutations cause a multisystem disorder by impairing secretion of COL4A1/A2 heterotrimers. We define parameters for reducing stroke and myopathy in *Col4a1* mutant mice by pharmacologically promoting heterotrimer secretion.

## INTRODUCTION

Collagen type IV alpha 1 (COL4A1) and alpha 2 (COL4A2) form heterotrimers that constitute one of the most abundant constituents of nearly all basement membranes. *COL4A1* and C*OL4A2* mutations cause a multisystem disorder characterized by the presence of cerebrovascular disease with variable ocular, renal and muscular involvement (Jeanne and Gould, 2017; Mao et al., 2015). The spectrum of cerebrovascular manifestations reported in individuals with *COL4A1* mutations includes porencephaly, perinatal and age-related intracerebral hemorrhages (ICHs), cerebral microbleeds and white matter abnormalities (Meuwissen et al., 2015; van der Knaap et al., 2006; Weng et al., 2012; Yoneda et al., 2012). In addition to Mendelian cases of cerebrovascular disease caused by rare *COL4A1* and *COL4A2* mutations, large-scale genetic studies identified associations between *COL4A1* and intracranial aneurysms, deep ICHs, lacunar ischemic stroke, reduced white matter volume, arterial calcification, arterial stiffness and leukoencephalopathy (Adi et al., 2014; Ayrignac et al., 2015; Di Donato et al., 2014; Livingston et al., 2011; O'Donnell et al., 2011; Rannikmae et al., 2015; Ruigrok et al., 2006; Tarasov et al., 2009). Moreover, *COL4A2* was associated with ICHs and white matter hyperintensities in stroke patients and ‘community populations’, suggesting that susceptibility factors are shared between stroke patients and the general population (Rannikmae et al., 2015, 2017; Traylor et al., 2016). In addition to the high prevalence of cerebrovascular disease in patients with *COL4A1* mutations, myopathy has been reported in over one-third of cases (Jeanne and Gould, 2017). The spectrum of muscular manifestations caused by *COL4A1* mutations is not well defined, but includes hypotonia, cramps and elevated serum creatine kinase levels (Labelle-Dumais et al., 2011; Plaisier et al., 2010, 2007). Despite a growing recognition for the roles of *COL4A1* and *COL4A2* mutations in the etiology of cerebrovascular disease and myopathy, there are currently no targeted therapeutic interventions.

COL4A1 and COL4A2 assemble into heterotrimers in the endoplasmic reticulum before being secreted into the extracellular matrix (Mayne et al., 1984; Trueb et al., 1982). The primary consequence of *COL4A1* and *COL4A2* mutations is impaired secretion of COL4A1/A2 heterotrimers (Gould et al., 2007, 2005; Jeanne et al., 2012; Kuo et al., 2014), and we previously demonstrated an inverse correlation between heterotrimer secretion efficiency and ICH severity in an allelic series of *Col4a1* mutant mice (Jeanne and Gould, 2017; Jeanne et al., 2015; Kuo et al., 2014). Sodium 4-phenylbutyrate (4PBA) is a US Food and Drug Administration-approved drug with chemical chaperone properties (Iannitti and Palmieri, 2011; Perlmutter, 2002; Rubenstein and Zeitlin, 1998) that effectively promoted secretion of mutant heterotrimers *in vitro* and *in vivo* and reduced ICH severity in *Col4a1* mutant mice, supporting the therapeutic potential of improving heterotrimer secretion for patients with *COL4A1* and *COL4A2* mutations (Jeanne et al., 2015; Kuo et al., 2014; Murray et al., 2014). Because there are few available treatment options for patients suffering from ICH, prevention is of the utmost importance. Here, we used different 4PBA administration paradigms in *Col4a1* mutant mice to define parameters for future interventions aimed at preventing, reducing or delaying cerebrovascular and muscular manifestations in patients with *COL4A1* or *COL4A2* mutations.

## RESULTS

### 4PBA treatment suppresses age-related and exercise-induced ICH in *Col4a1^+/Δex41^* mice

**Fig. 1.**
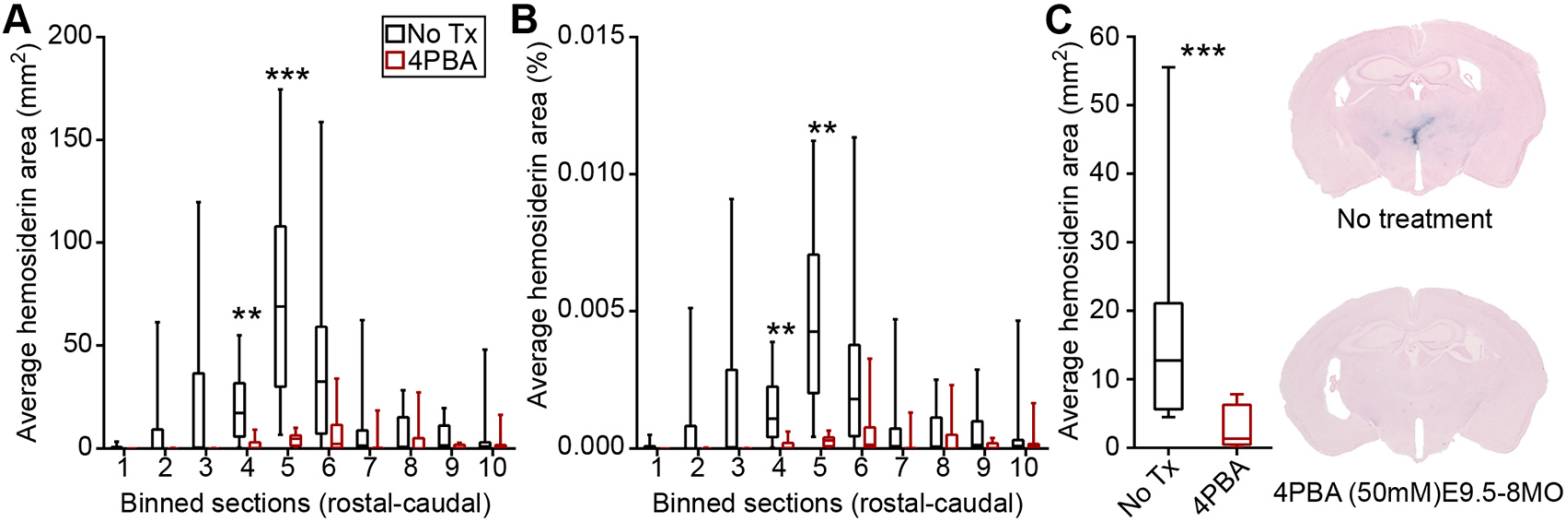
**Long-term 4PBA treatment ameliorates ICH in *Col4a1^+/Δex41^* mice.** *Col4a1^+/Δex41^* mice were treated with 50 mM 4PBA continuously from E9.5 to 8MO. (A,B) ICH was predominantly observed in subcortical regions corresponding to bins four to six (+0.14 mm to −2.8 mm from bregma), when expressed as absolute area (A) and as a percentage relative to the size of the brain sections (B). (C) Average hemosiderin values across the whole brain for each animal show significantly reduced ICH severity in 4PBA-treated mice. Significance was determined by two-tailed Mann–Whitney test and multiple Student's *t*-test, assuming unequal variance with Holm–Sidak correction. Box and whisker plots show median, interquartile range, and maximum and minimum values (*n*=10, **P<0.01, ***P<0.001).

**Fig. 2.**
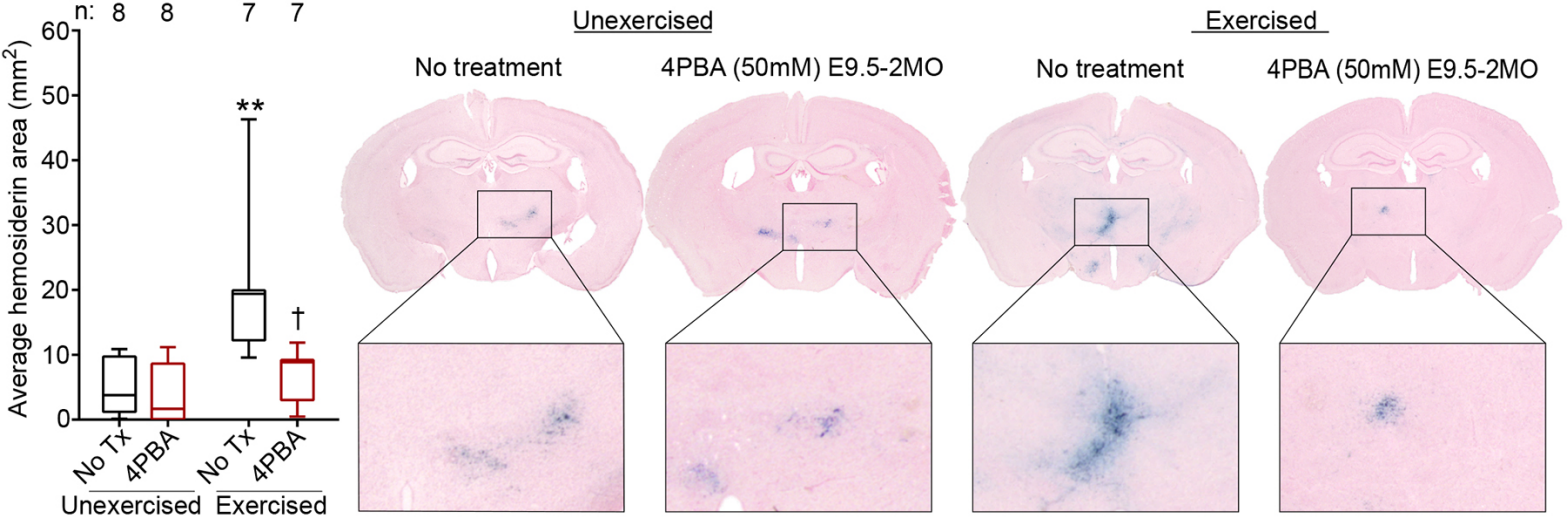
**4PBA treatment significantly reduces ICH severity in exercised *Col4a1^+/Δex41^* mice.** There was no difference in ICH severities between *Col4a1^+/Δex41^* mice provided with 50 mM 4PBA from E9.5 to 2MO and their untreated counterparts. However, ICH severity was exacerbated by exercise challenge and this was significantly reduced by 4PBA treatment from E9.5 to 2MO. Statistical analyses were performed using Kruskal–Wallis test with Dunn's multiple comparison test. Box and whisker plots show median, interquartile range, and maximum and minimum values. Sample sizes are indicated in the figure; **P<0.01, compared with the unexercised, untreated cohort; ^†^*P*<0.05, compared with the exercised, untreated cohort.

Mice heterozygous for a *Col4a1* splice site mutation that causes skipping of exon 41 (*Col4a1^+/Δex41^*) recapitulate the pathophysiological hallmarks of cerebrovascular and muscular disease observed in patients with *COL4A1* and *COL4A2* mutations, and thus constitute a powerful pre-clinical model to test the efficacy and treatment parameters of potential therapeutic agents (Gould et al., 2005, 2006; Kuo et al., 2012). Cerebrovascular disease is a particularly devastating consequence of *COL4A1* mutations, and we have previously demonstrated that ICH severity in *Col4a1^+/Δex41^* mice increases with age and is exacerbated by environmental factors, such as birth trauma, exercise and anticoagulants (Gould et al., 2006; Jeanne et al., 2015). In addition, we previously showed that pharmacologically promoting heterotrimer secretion reduced ICH caused by *COL4A1* mutation in young mice (Jeanne et al., 2015). To test whether long-term administration of 4PBA could suppress age-related ICH in *Col4a1^+/Δex41^* mice, we provided *Col4a1^+/+^* and *Col4a1^+/Δex41^* littermates with 50 mM 4PBA in drinking water from embryonic day (E) 9.5 to 8 months old (MO) and measured ICH severity using Perls' Prussian Blue staining. This is an efficacious and well-tolerated dose (Rubenstein and Zeitlin, 1998), and we confirmed 4PBA bioavailability in embryos, nursing pups and weaned mice (Table 1). In all experiments described in this study, no differences were observed between males and females, and pathology was never detected in *Col4a1^+/+^* mice (not shown). In *Col4a1^+/Δex41^* mice, ICH was predominantly observed in a region of the basal ganglia located between +0.14 mm and −2.8 mm from bregma (Fig. 1A,B). Importantly, ICH severity was significantly reduced in 8MO 4PBA-treated *Col4a1^+/Δex41^* mice compared with their untreated counterparts (Fig. 1C), demonstrating the efficacy of long-term 4PBA treatment in suppressing ICH in *Col4a1^+/Δex41^* mice. Next, we repeated this experiment (50 mM 4PBA starting at E9.5) in mice aged up to 2MO with or without exercise challenge. In unexercised cohorts, untreated *Col4a1^+/Δex41^* mice had relatively mild ICH, and we did not detect differences between treated and untreated *Col4a1^+/Δex41^* mice. Exercise challenge significantly exacerbated ICH in untreated 2MO *Col4a1^+/Δex41^* mice and this was prevented by 4PBA treatment (Fig. 2).

**Table 1.**
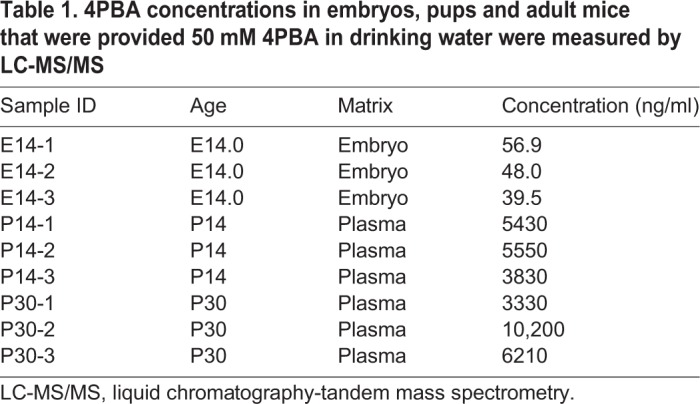
**4PBA concentrations in embryos, pups and adult mice that were provided 50 mM 4PBA in drinking water were measured by LC-MS/MS**

### Early transient 4PBA administration has sustained protective effects

**Fig. 3.**
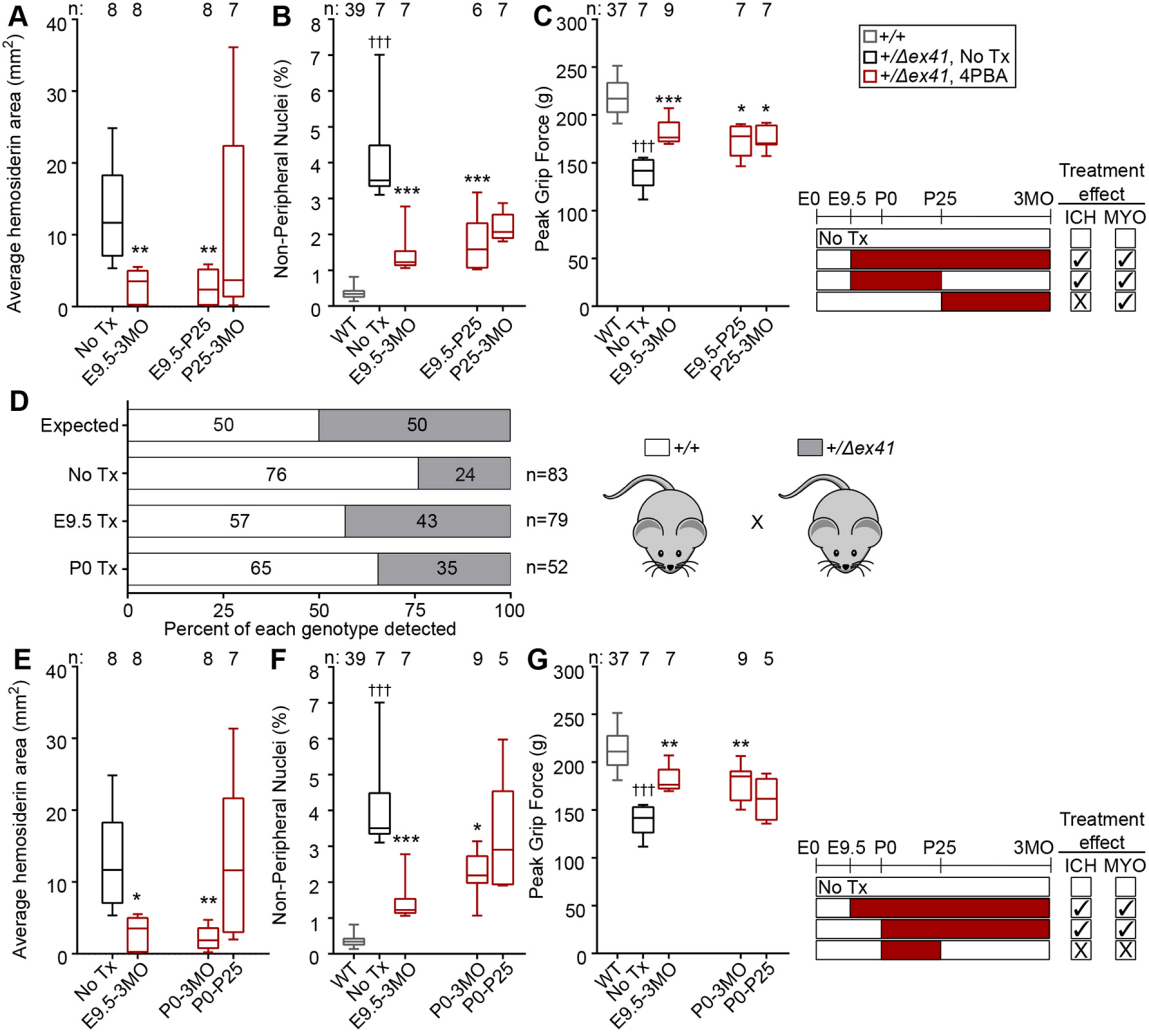
**Evaluation of temporal parameters for treatment of ICH and myopathy in *Col4a1^+/Δex41^* mice.** Mice were provided with 50 mM 4PBA for varying time intervals as depicted on the timeline schematics. (A-C) Providing *Col4a1^+/Δex41^* mice with 4PBA prenatally was more effective at suppressing ICH and myopathy (reduced NPN, increased grip strength) than providing 4PBA after weaning, and appeared to have sustained benefits [NPN and grip force for *Col4a1^+/+^* mice were indistinguishable among all treatment groups and were pooled as wild type (WT) in the graphs]. (D) Comparing the proportion of *Col4a1^+/+^* and *Col4a1^+/Δex41^* littermates at weaning revealed that viability of *Col4a1^+/Δex41^* mice was preserved in mice treated with 4PBA prenatally. (E-G) Postnatal 4PBA treatment must be provided continuously to suppress ICH, reduce NPN and increase grip strength in *Col4a1^+/Δex41^* mice. Data points for WT, untreated *Col4a1^+/Δex41^* mice and *Col4a1^+/Δex41^* mice treated with 4PBA from E9.5 to 3MO are included in multiple panels to facilitate comparisons between treatment paradigms. Statistical analyses were performed using Kruskal–Wallis test with Dunn's multiple comparison test (A-C,E-G). Box and whisker plots show median, interquartile range, and maximum and minimum values. Sample sizes are indicated in the figure; *P<0.05, **P<0.01, ***P<0.001, compared with No Tx; ^†††^*P*<0.001, compared with WT. The distribution of genotype frequencies (D) was analyzed by Fisher's exact test compared with expected; *P*<0.001, *P*=0.43 and *P*=0.16 for No Tx, E9.5 Tx and P0 Tx, respectively.

To define potential interventional windows, we treated *Col4a1^+/+^* and *Col4a1^+/Δex41^* littermates with 50 mM 4PBA for varying time intervals. We aged mice to 3MO, challenged them with exercise and extended the analyses to test the effects of 4PBA on skeletal myopathy. To this end, we measured grip strength to evaluate muscle function and then challenged the mice with exercise 24 h prior to assessment of ICH severity by Perls' Prussian Blue and muscle damage by counting nonperipheral nuclei (NPN) in quadriceps muscles. ICH severity in untreated *Col4a1^+/Δex41^* mice was similar to that observed in untreated *Col4a1^+/Δex41^* mice at 2MO (exercised) and 8MO (unexercised), and both ICH and skeletal myopathy were significantly suppressed in *Col4a1^+/Δex41^* mice provided with 4PBA from E9.5 to 3MO (Fig. 3A-C). Importantly, this treatment paradigm also significantly reduced the peri-/postnatal lethality that is characteristic of *Col4a1^+/Δex41^* mice (Gould et al., 2005) (Fig. 3D). To test the relative impact of early versus late intervention, we evaluated ICH and myopathy severity in *Col4a1^+/Δex41^* mice for which 4PBA was either discontinued or initiated at weaning [treated from E9.5 to postnatal day (P) 25 or from P25 to 3MO, respectively]. ICH severity in *Col4a1^+/Δex41^* mice with discontinued 4PBA administration (E9.5 to P25) was significantly reduced to levels that were indistinguishable from *Col4a1^+/Δex41^* mice that received continuous 4PBA from E9.5 to 3MO (Fig. 3A). In contrast, when 4PBA was initiated at P25, there was no significant difference in ICH severity between the untreated and treated cohorts (Fig. 3A). Myopathy severity in 3MO *Col4a1^+/Δex41^* mice was significantly reduced, irrespective of whether 4PBA treatment was discontinued or initiated at P25 (Fig. 3B,C). Collectively, these findings suggest that different pathologies might have different therapeutic windows and that early 4PBA administration can provide sustained protection, even after it is discontinued.

### Postnatal 4PBA reduces ICH and skeletal myopathy in *Col4a1^+/Δex41^* mice

Because prenatal detection of *COL4A1* mutations is uncommon and embryonic intervention unlikely, postnatal treatment windows are more relevant for clinical interventions*.* To further define postnatal treatment opportunities, we provided mice with 50 mM 4PBA from birth (P0) to 3MO. Grip strength was measured and mice were subjected to an exercise challenge 24 h prior to ICH and NPN quantification. Compared with their untreated counterparts, ICH severity was significantly lower in *Col4a1^+/Δex41^* mice provided with 4PBA from P0 to 3MO, and was comparable to that of *Col4a1^+/Δex41^* mice treated from E9.5 to 3MO (Fig. 3E), with one important distinction – postnatal treatment did not prevent peri-/postnatal lethality (Fig. 3D). Myopathy was also ameliorated in *Col4a1^+/Δex41^* mice treated from P0 to 3MO, but to a lesser extent than the cohort treated from E9.5 (Fig. 3F,G). When transient 4PBA treatment was provided postnatally from P0 to P25, the severities of ICH and myopathy were not significantly different between treated and untreated *Col4a1^+/Δex41^* mice (Fig. 3E-G). This is in contrast to the sustained benefits of transient 4PBA administration provided from E9.5 to P25 (Fig. 3A-C). Together, these findings underscore the importance of early intervention and suggest that postnatal treatment can be effective in reducing *COL4A1*-related ICH and skeletal myopathy when it is not discontinued.

### Dose-dependent responses to 4PBA

**Fig. 4.**
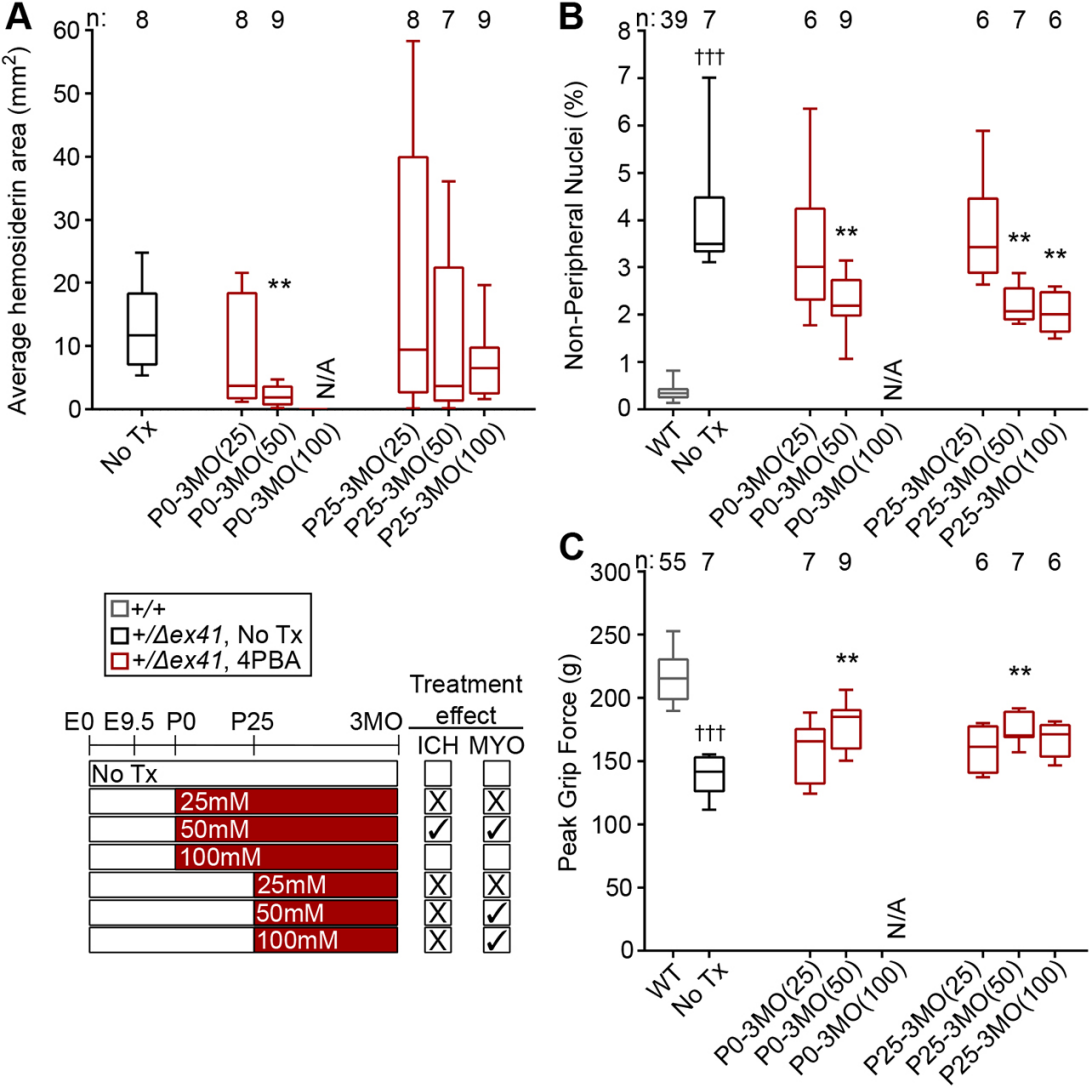
**Evaluation of 4PBA dose dependency for postnatal treatment of ICH and myopathy in *Col4a1^+/Δex41^* mice.** *Col4a1^+/Δex41^* mice were treated with 25 mM, 50 mM or 100 mM 4PBA from birth (P0) or weaning (P25). NPN and grip force for *Col4a1^+/+^* mice were indistinguishable among all treatment groups and were pooled as WT in the graphs. (A) Evaluation of 4PBA dose dependency suggested that 4PBA might be effective in a specific concentration range, whereby high levels can have detrimental effects on pregnancy and nurturing behavior, and low levels can exacerbate ICH. (B,C) Dose dependency was also observed for muscle parameters [reduced NPN (B) and increased grip strength (C)]. Data points for WT, untreated *Col4a1^+/Δex41^* mice and *Col4a1^+/Δex41^* mice treated with 4PBA from E9.5 to 3MO are included in multiple panels to facilitate comparisons between treatment paradigms. Statistical analyses were performed using Kruskal–Wallis test with Dunn's multiple comparison test. Box and whisker plots show median, interquartile range, and maximum and minimum values. Sample sizes are indicated in the figure; *P<0.05, **P<0.01, ***P<0.001, compared with No Tx; ^†††^*P*<0.001, compared with WT.

Finally, we tested 4PBA dose dependency on ICH and skeletal myopathy severities in mice that received continuous postnatal treatment. To this end, we provided *Col4a1^+/Δex41^* mice with 25 mM, 50 mM or 100 mM 4PBA from birth (P0) or weaning (P25) until assessment of ICH and myopathy at 3MO, 24 h after grip strength measurement and exercise challenge. Dams provided with 100 mM 4PBA failed to nurture their pups, precluding further analysis of the cohort treated from P0. As noted earlier, providing *Col4a1^+/Δex41^* mice with 50 mM 4PBA from birth significantly suppressed ICH severity, reduced the number of NPN and increased grip strength (Fig. 4A-C). Notably, ICH and myopathy in approximately half of the *Col4a1^+/Δex41^* mice from the cohort provided with 25 mM 4PBA from birth were milder than the mildest case of the untreated *Col4a1^+/Δex41^* cohort; however, the population showed broad ranges of severities (Fig. 4A-C). When 4PBA treatment was initiated at weaning (P25), we also observed a trend toward dose dependency for ICH, whereby outcomes appeared to improve with increasing concentration (Fig. 4A); however, no additional benefits of 100 mM 4PBA were detected for myopathy when compared with 50 mM 4PBA (Fig. 4B,C). Strikingly, the poor ICH outcome in the 25 mM (and to some extent the 50 mM) 4PBA cohort treated from P25 appears to be driven by a subset of mice with ICH that was more severe than that observed in the *Col4a1^+/Δex41^* mice that never received treatment. Together, these data suggest that the therapeutic benefits of 4PBA might be limited to a specific concentration range and that 4PBA administration at doses below a certain threshold could exacerbate ICH.

## DISCUSSION

*COL4A1* and *COL4A2* mutations are well-established causes of variable cerebrovascular and muscle diseases (Ayrignac et al., 2015; Di Donato et al., 2014; Meuwissen et al., 2015; Rannikmae et al., 2015; Ruigrok et al., 2006; Traylor et al., 2016; van der Knaap et al., 2006; Weng et al., 2012; Yoneda et al., 2012) with no specific intervention available to patients. The pathogenesis of *COL4A1*-related ICH and myopathy is thought to result from impaired heterotrimer secretion into basement membranes, and we previously demonstrated the therapeutic potential of promoting secretion using 4PBA administration in *Col4a1^+/Δex41^* mice. Here, we used different 4PBA treatment paradigms in *Col4a1* mutant mice to define the therapeutic parameters of promoting heterotrimer secretion for treatment of *COL4A1*-related ICH and myopathy. We identified therapeutic windows for alleviating ICH and myopathy and demonstrate a dose dependency for disease outcomes in response to postnatal 4PBA treatment in *Col4a1* mutant mice.

We chose to deliver 4PBA via drinking water in order to initiate treatment embryonically and allow continuous long-term administration to identify clinically relevant windows for intervention. Using continuous 4PBA administration from mid-embryogenesis, we show the long-term efficacy of promoting heterotrimer secretion in suppressing ICH in *Col4a1* mutant mice aged to 8MO. Importantly, ICH was predominantly detected in the basal ganglia of 8MO *Col4a1* mutant mice, which contains perforating vessels that are commonly compromised in cerebral small vessel disease (Gould et al., 2006; Pantoni, 2010). This observation suggests that selective sampling of the critical anatomical regions (as opposed to the entire brain) might represent a more efficient approach to test the efficacy of potential therapeutic interventions in reducing ICH in this pre-clinical model.

Unexpectedly, although we previously showed reduced ICH severity in 1MO *Col4a1^+/Δex41^* mice that received intermittent intragastric and intraperitoneal 4PBA injections (Jeanne et al., 2015), we did not detect an effect on ICH in 2MO *Col4a1* mutant mice when 4PBA was administered continuously via drinking water unless they were challenged by exercise. One possible explanation for this observation is that the route of 4PBA administration might influence its efficacy. Alternatively, the distinct developmental time course and broad distribution of microbleeds versus larger subcortical hemorrhages in the basal ganglia of *Col4a1* mutant mice could account for this discrepancy (Ratelade et al., 2018). Indeed, multifocal microbleeds are typically observed throughout the brains of newborn and juvenile mutant animals and appear to resolve by 3 months of age, whereas the onset of subcortical hemorrhages in the basal ganglia of *Col4a1* mutant mice generally occurs in early adulthood and progressively increase with age (Gould et al., 2005; Jeanne et al., 2015; Ratelade et al., 2018). Thus, it is possible that early cerebral microbleeds detected in 1MO animals are largely cleared by 2MO, while the incidence of subcortical hemorrhage only becomes prominent after 2MO or in response to environmental triggers such as exercise.

Using different treatment paradigms, we show that the maximal therapeutic benefits of 4PBA for suppressing *Col4a1*-related ICH and myopathy are achieved when treatment is initiated prenatally. Moreover, we demonstrate that transient 4PBA treatment initiated prenatally has sustained beneficial effects even after treatment was discontinued. Collectively, these data are consistent with a role for COL4A1 during development and underscore the importance of early intervention (Jeanne et al., 2015; Poschl et al., 2004). However, our results show that postnatal 4PBA treatment can also reduce ICH and myopathy, which has important clinical implications as therapeutic intervention in patients is unlikely to be initiated during gestation. Interestingly, initiating treatment after weaning significantly suppressed myopathy but not ICH, raising the possibility that different pathologies might also have different interventional timeframes.

Although our findings clearly demonstrate the therapeutic potential of pharmacologically promoting heterotrimer secretion to reduce *COL4A1*-related ICH and myopathy, they also highlight important limitations when considering the translational potential of this approach. For instance, when postnatal treatment was discontinued, the therapeutic benefit appeared to be lost. We also identified a postnatal dose dependency in the response to 4PBA treatment, whereby increasing variability in ICH severity was observed at lower 4PBA concentrations. This variability appears to be driven by a subset of mice that experience worse outcomes than untreated mice. It is unclear whether this effect is specific to 4PBA or a general consequence of promoting heterotrimer secretion that might also manifest with other chemical chaperones.

Collectively, our findings show that maximal benefits of pharmacologically promoting heterotrimer secretion for prevention of *COL4A1*-related ICH and myopathy require prenatal intervention, but that postnatal treatment has therapeutic potential, which has clinical implications for patients with *COL4A1* and *COL4A2* mutations. However, the possibility that low 4PBA concentrations exacerbate ICH in mice with a *Col4a1* mutation underscore that caution is necessary for designing clinical trials to identify therapeutic interventions to prevent, reduce or delay pathology caused by *COL4A1* and *COL4A2* mutations. Further work is required to determine whether chemical chaperones represent a viable approach and highlight the importance of identifying more specific therapeutic agents for *COL4A1*-related cerebrovascular and muscle diseases.

## MATERIALS AND METHODS

### Animals

All experiments were conducted in compliance with protocols approved by the UCSF Institutional Animal Care and Use Committee (protocols AN102193 and AN159737). *Col4a1^+/Δex41^* mice and *Col4a1^+/+^* littermates were maintained on a C57BL/6J background (*N*>20) and weaned at P25 owing to their small size. Both male and female mice were used in all experiments and no differences were observed between sexes. Samples were not excluded in this study.

### 4PBA treatment

Sodium 4-phenylbutyrate (Scandinavian Formulas Inc., Sellesville, PA, USA) was provided in drinking water and refreshed weekly. E9.5 was chosen to avoid potential implantation perturbations and minimize possible teratogenic effects. The day of fertilization (E0) was assessed by the presence of a vaginal plug.

### Exercise challenge

Mice were exercise challenged on a treadmill in a single session 24 h prior to harvesting for the 3MO time-point, or a series of five sessions performed 3 days apart from the 2MO time-point. Each exercise session included a 2-min acclimation period, followed by a 30-min exercise challenge with a 15° downhill grade on a treadmill equipped with a shock plate (Exer 3/6, Columbus Instruments, Columbus, OH, USA). Animals were started at 7 m/min and increased by 3 m/min every 2 min until a maximum speed of 12 m/min was reached (Jeanne et al., 2015).

### Perls’ Prussian Blue staining and analysis

Tissue preparation and Perls' Prussian Blue staining was described previously (Jeanne et al., 2015). Unbiased analysis of hemosiderin area was performed using CellProfiler (Lamprecht et al., 2007). We separated color images based on absorbance composition to segregate image features such as Perls' Prussian Blue, fresh hemorrhage and total tissue area. We identified the total brain area of a section and overlaid the mask on the Perls' Prussian Blue area and fresh hemorrhage area, and identified signal from the masked region. Pixel counts of features were measured and converted to area in mm^2^. To evaluate ICH distribution, we divided the rostro-caudal axis into 10 bins consisting of six sections spanning ∼1 mm.

### Quantification of nonperipheral nuclei

Immediately after dissection, quadriceps muscles were flash frozen in liquid nitrogen-chilled isopentane. Cryosections (10 µm) were collected from the central portion of the muscles at regular intervals (200 µm) and stained with Hematoxylin and Eosin for histopathological analysis and determination of the numbers of NPN. Between 16 and 20 sections were examined per muscle and one random field of view (20×) was imaged for each section for subsequent NPN quantification (Labelle-Dumais et al., 2011).

### Quantification of 4PBA *in vivo*

Liquid chromatography tandem-mass spectrometry was performed by Quintara Biosciences (South San Francisco, CA, USA). Plasma (10 μl) or embryo homogenate (20 μl) were treated with 100 μl methanol:acetonitrile (1:1 v:v) containing 50 ng/ml of internal standard tolbutamide. The samples were vigorously vortexed for 25 min and centrifuged for 15 min at 4000 rpm (1500 ***g***) before reconstituting 50 μl of the extract with 70 μl water. The calibration standards of 4PBA were prepared by spiking the compound into the corresponding plasma/embryo blank matrix and processed in the same way as the samples. The analysis used negative electrospray ionizations under the multiple-reaction-monitoring mode for the detection of samples and the internal standard.

### Statistical analyses

Power calculations for ICH, NPN and grip strength were performed for 3MO mice using R pwr-package (https://cran.r-project.org/web/packages/pwr/index.html) with power of 0.9 and significance set to 0.05. Minimal sample sizes for ICH, NPN and grip strength were 7, 3 and 3, respectively. Statistical analyses were performed using GraphPad Prism (GraphPad Software, Inc., La Jolla, CA, USA). For two-group comparisons, we used two-tailed Mann–Whitney test and multiple Student's *t*-test assuming unequal variance with Holm–Sidak correction. For multiple-group comparisons, we used Kruskal–Wallis test (one-way ANOVA) with Dunn's multiple comparison test. Values of *P*<0.05 were considered statistically significant. Data are presented as box and whisker plots representing the median, interquartile range and maximum range. We used Fisher's exact test to compare observed and expected frequencies of mutant and wild-type littermates.
